# Head-to-head comparison of diffusion imaging markers for cognitive decline and risk of dementia: The population-based Rotterdam Study

**DOI:** 10.1162/IMAG.a.1382

**Published:** 2026-10-06

**Authors:** Mathijs T. Rosbergen, Frank J. Wolters, M. Arfan Ikram, Meike W. Vernooij

**Affiliations:** Department of Epidemiology, Erasmus MC – University Medical Center Rotterdam, Rotterdam, the Netherlands; Department of Radiology & Nuclear Medicine and Alzheimer Centre, Erasmus MC – University Medical Center Rotterdam, Rotterdam, the Netherlands

**Keywords:** dementia, Alzheimer’s disease, MRI, neuroimaging, diffusion tensor imaging (DTI)

## Abstract

Diffusion tensor imaging (DTI) is a promising tool for early detection of white matter deterioration, but the optimal DTI marker for assessing risk of cognitive decline and dementia is undetermined. The aim of this study was to compare the predictive value of multiple diffusion measures for cognitive decline and dementia risk in a population-based study. We measured peak-width skeletonized mean diffusivity (PSMD), normal-appearing white matter mean diffusivity (MD), and fractional anisotropy (FA) in participants aged 45 years and older who underwent 1.5T MRI between 2006–2015 as part of the ongoing prospective population-based Rotterdam Study. In a head-to-head comparison between DTI markers, we determined risk of cognitive decline and dementia during follow-up, using linear regression and Cox proportional hazard models, respectively, that also include age, sex, education, and presence of lacunes or microbleeds. Of 5230 participants (mean age: 65.0 years +/-10.9 [standard deviation], 55.8% women), 286 developed dementia during a median follow-up of 9.8 years. For dementia risk, PSMD and global MD were superior regarding model fit, with the highest hazard ratio for global MD (HR: 1.74 [95%CI: 1.53–1.98])*.* Similarly, PSMD and MD had the highest discriminatory value (C-statistic, for PSMD: 0.886, and for MD: 0.882), albeit differences with other measures were small. Of all participants, 4433 (84.3%) had complete data on all cognitive tests at baseline, of whom 2800 (63.2%) completed their follow-up visit after on average 5.6 years. For global cognition both at time of MRI and at follow-up, PSMD was superior in terms of model fit and showed the highest regression coefficients, followed by tract-specific measures of MD and FA. In conclusion, when directly comparing structural connectivity measures, PSMD is superior to MD and FA for prediction of cognition and cognitive decline, while PSMD and MD perform best with respect to dementia risk.

## 1 Introduction

Dementia represents significant and growing public health challenges, with an increasing burden on aging populations worldwide (Aranda et al., 2021). Cognitive decline and dementia are characterized by progressive loss of cognitive abilities such as memory, attention, and executive function, which are due, in part, to deterioration of white matter networks (Charlton et al., 2006). Quantitative assessment of this white matter injury in an early stage could improve timely identification of individuals at risk of cognitive decline and dementia. Although white matter hyperintensities (WMH) volume on conventional magnetic resonance imaging (MRI) techniques is predictive of future cognitive decline and dementia (Hu et al., 2021), it reflects only the tip of the iceberg of white matter injury (Rosbergen et al., 2024). Diffusion tensor imaging (DTI) has emerged as a key modality for assessing white matter integrity in a more fine-grained manner (Alexander et al., 2007), detecting microstructural changes in normal-appearing white matter (NAWM) years before they manifest as WMH (De Groot et al., 2013). As such, DTI measures are a promising marker for early risk stratification, but implementation of DTI in clinical practice is limited, due, in part, to uncertainty about the optimal DTI parameters for assessment of risk of cognitive decline.

Of the commonly used DTI metrics, fractional anisotropy (FA) and mean diffusivity (MD) have demonstrated associations with cognitive decline and dementia (Charlton et al., 2006; Cremers et al., 2016, 2020), but they are limited by their susceptibility to noise and artifacts. A more recent diffusion imaging marker, peak-width skeletonized mean diffusivity (PSMD), has gained attention for its ability to robustly quantify microstructural changes in white matter (Baykara et al., 2016). Instead of averaging diffusivity values, PSMD quantifies the width of the distribution of diffusivity values across the white matter skeleton. The width of the skeletonized MD histogram may be more sensitive to early, localized damage in the white matter than the global MD value. Moreover, PSMD is fully automated and standardized, ensuring highly reproducible across different scanners and settings (Maillard et al., 2022), which enhances its potential for widespread clinical application. Several studies have explored the relationship between PSMD and cognitive decline (Deary et al., 2019; Lam et al., 2019; Luckey et al., 2024), or dementia risk (Jacob et al., 2023), but none directly compared the prognostic value of PSMD to conventional diffusion imaging markers, such as FA and MD.

The aim of this study was to provide a head-to-head comparison of the predictive value of multiple diffusion measures for cognitive decline and dementia risk in a population-based study.

## 2 Methods

### 2.1 Study population

This research was embedded in the Rotterdam Study, an ongoing prospective population-based cohort in the Netherlands (Ikram et al., 2024) that started in 1990, enrolling residents aged ≥45 years in the Ommoord district in Rotterdam with follow-up every 4 to 6 years. Participants overlapped with prior DTI studies (Cremers et al., 2016, 2020), but we focused on directly comparing the predictive value of DTI markers, including PSMD. However, of the 5913 participants who underwent brain MRI, we included 5230 participants with brain MRI between March 29, 2006, and September 22, 2015 (Fig. 1). The Rotterdam Study received approval from the institutional review board of the Erasmus Medical Center and by the Ministry of Health, Welfare and Sports of the Netherlands, implementing the “Wet Bevolkingsonderzoek: ERGO (Population Screening Act: Rotterdam Study).” All participants provided written informed consent for their participation in the study.

**Fig. 1.**
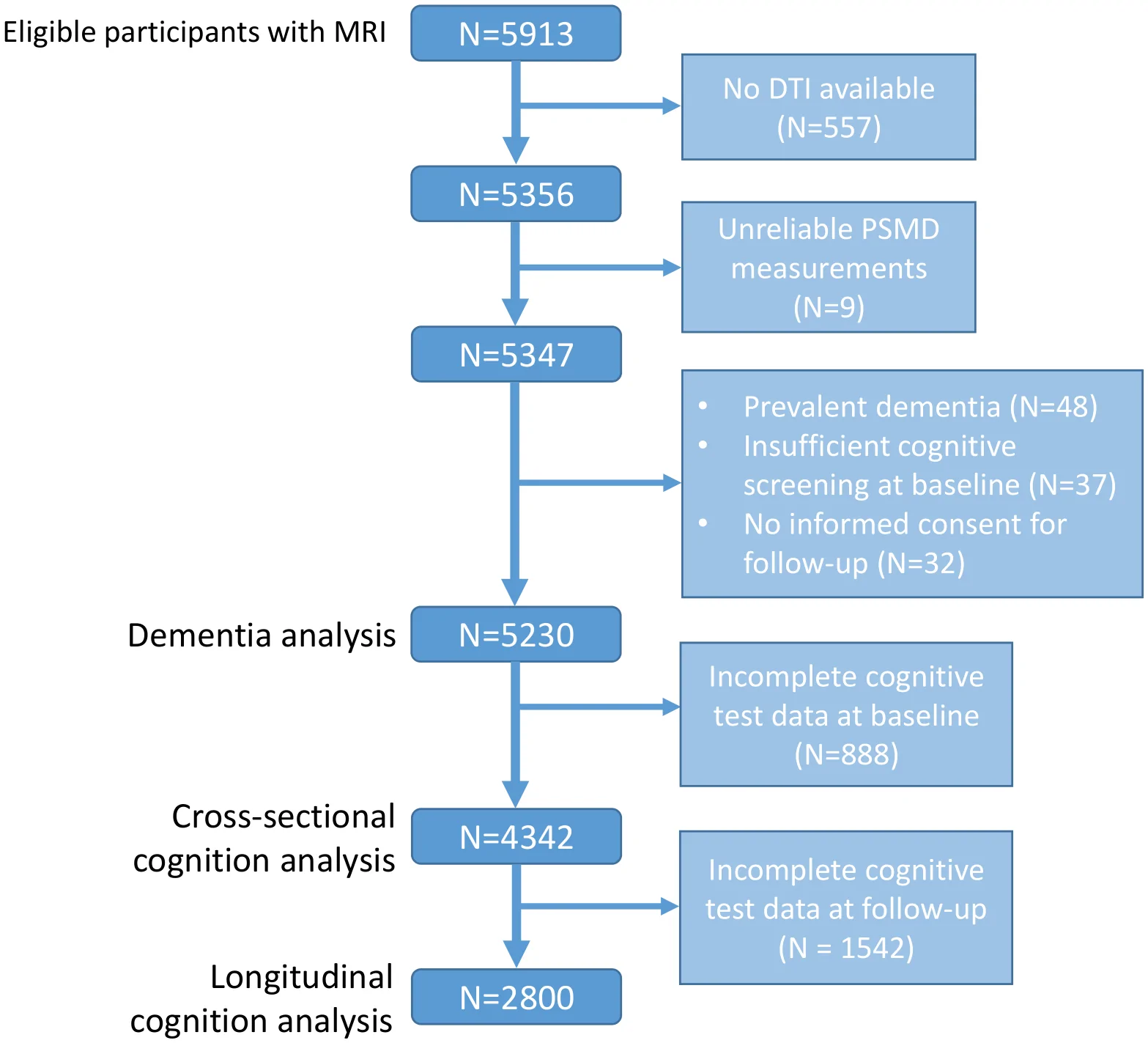
Flowchart of the inclusion of participants.

### 2.2 MRI acquisition, preprocessing, and appraisal

All participants underwent brain MRI using a 1.5 Tesla MRI scanner (Signa Excite II; General Electric Healthcare) with an 8-channel head coil. All scans were acquired using a single scanner in which no changes in hardware, and software took place. The scan protocol and sequence details of the Rotterdam Study have been described elsewhere (Ikram et al., 2024, 2015). For volumetry, T1-weighted, proton density-weighted, and FLAIR scans were used for automated segmentation of supratentorial white matter, WMH, and intracranial volume (De Boer et al., 2009; Vrooman et al., 2007). Normal-appearing white matter (NAWM) was defined as white matter with WMH excluded. Two raters visually inspected and manually corrected the segmentations if necessary.

For diffusion tensor imaging, we performed a single-shot, diffusion‐weighted spin echo‐planar imaging sequence to quantify global FA and global MD of NAWM (repetition time [TR] = 8000 milliseconds, echo time [TE] = 68.7 milliseconds, field of view [FOV] = 21 × 21 cm2, matrix = 96 × 64 [interpolated to 128 × 128], slice thickness = 3.5 mm, 36 contiguous slices, applying parallel imaging with acceleration factor = 2, acquired voxel size = 2.2 × 3.3 × 3.5 mm3, interpolated voxel size = 1.6 × 1.6 × 3.5 mm3). Maximum b‐value was 1000 seconds/mm^2^ in 25 noncollinear directions (number of excitations (NEX) = 1), and three volumes were acquired without diffusion weighting (b ‐value = 0 second/mm^2^). Data were processed using a standardized pipeline (Koppelmans et al., 2014) and including only voxels with FA ≥ 0.2 to minimize partial volume effects (Koppelmans et al., 2014). Eddy current and head-motion correction were performed on the diffusion data. The resampled data were used to fit diffusion tensors. PSMD was derived using an automated pipeline (v0.95) (Baykara et al., 2016) and the white matter skeleton (Smith et al., 2006). FA values ≥ 0.2 and subsequently MD values were projected on this skeleton. PSMD was then calculated as the difference between the 95th and 5th percentiles of voxel-based MD values. MRI scans with unreliable PSMD values (>0.0007) were excluded based on previous research (Beaudet et al., 2020). On top of global FA, global MD, and PSMD, we included tract-specific FA and tract-specific MD measures (de Groot et al., 2015) of the inferior front-occipital fasciculus, and the posterior thalamic radiation, as they were the most strongly related to poor cognition in previous research (Cremers et al., 2016). For these white matter tracts, we combined measures from the left and right hemisphere to obtain a summary measurement for each tract (de Groot et al., 2015).

All scans were rated by trained researchers, blinded for clinical data, for the presence of lacunes and microbleeds. Lacunes were defined as focal parenchymal lesions ranging from ≥3 mm to <15 mm in size with similar signal intensity as CSF on all sequences, and—when located supratentorial—with a hyperintense rim on FLAIR images (Vernooij et al., 2007). To differentiate lacunes from dilated perivascular spaces, symmetry of the lesions, sharp demarcation, and absence of a hyperintense rim on the FLAIR sequence supported the presence of a dilated perivascular space (Adams et al., 2013). Microbleeds were identified as focal areas with a very low intensity on a 3D T2*-weighted gradient-recalled echo MRI scan (voxel size 0.78 × 1.12 × 1.6 mm3), unaccompanied by signal abnormalities on other sequences (Ikram et al., 2015). Both presence of lacunes and presence of microbleeds were coded as binary variables (yes/no).

### 2.3 Educational attainment

Information regarding educational attainment was obtained during baseline home interviews and categorized into the following four categories: primary education, lower/intermediate vocational or lower vocational education, intermediate vocational education or higher general education, and higher vocational education or university.

### 2.4 Assessment of cognition

Assessment of cognitive function was routinely performed at center visits every 4 to 6 years through a cognitive test battery, including the color-word interference subtask of the Stroop test (Golden, 1976), measuring processing and attention, the Word Fluency test (WFT) (Welsh et al., 1994), measuring efficiency of searching long term memory, the Letter Digit Substitution test (LDST) (Lezak, 2004), measuring processing speed and executive function, the delayed recall score of the 15-word verbal learning test (15-WLT) (Lezak, 1984), measuring verbal learning and memory, and Purdue Pegboard test (PPB) (Desrosiers et al., 1995), measuring dexterity and fine motor speed. Additionally, we derived a global cognition measure (g-factor) from principle component analysis of these five cognition tests (Hoogendam et al., 2014), defined as the first unrotated component, explaining 49.8% of variance in cognition in our sample. The cognition tests had similar contribution to the derivation of the g-factor (loadings: LDST = 0.51; PPB = 0.43; Stroop = -0.46; WFT = 0.43. 15-WLT = 0.40). A higher g-factor indicates a better cognitive performance. Participants with incomplete data for all five cognitive tests were excluded.

### 2.5 Assessment of dementia

All participants were followed for the occurrence of dementia through a combination of repeated in-person assessment and linkage with electronic medical records. Participants were screened for dementia at baseline and every 3–6 years during follow-up examinations using the Mini-Mental State Examination (MMSE) and the Geriatric Mental State Schedule (GMS) organic level. Participants with a MMSE <26 or a GMS >0 underwent an examination and informant interview with the Cambridge Examination for Mental Disorders of the Elderly. A consensus panel, led by a neurologist, confirmed dementia diagnoses using DSM-III-R criteria. Participants were censored at dementia diagnosis, death, loss to follow-up or January 1, 2020, whichever occurred first.

### 2.6 Data analysis

Missing covariate data (≤1.0%) were imputed using 5-fold multiple imputation. We modeled age nonlinearity using natural cubic splines of age with 1 knot. All diffusion measures were standardized, and FA measures were inverted to ease comparison.

In our primary analysis, we used Cox proportional hazard models to obtain HRs and 95%CIs for the association between global DTI (PSMD, global MD, global FA) measures and risk of dementia. Additionally, we calculated the Akaike Criterium Information (AIC) and C-statistic. We included in all models age, sex, education, and the presence of lacunes and/or microbleeds. We then determined the cross-sectional association between global DTI measures and the g-factor, using linear regression models, including the same variables as before. For longitudinal models, we assessed the association between baseline global DTI measures and g-factor at the next follow-up visit, adjusting for baseline g-factor and follow-up time by adding these as covariates in our models.

In a post-hoc analysis, we assessed the C-statistic with both MD and PSMD included. Additionally, we compared the predictive value of white matter hyperintensities (WMH) volume for dementia and cognitive decline with that of the diffusion imaging markers.

In secondary analyses, we assessed tract-specific measures of FA and MD in relation to dementia risk and cognitive decline. Cognition analyses were repeated for each of the cognitive tests separately.

In a sensitivity analysis, we stratified dementia risk analyses by follow-up time. All analyses were performed using R, version 4.3.3 (packages: dplyr, ggplot2, mice, sjmisc, splines, survival). R codes can be found in this repository: https://github.com/MathijsR1/analysis-code-Head_to_head_comparison_of_diffusion_imaging_markers.git

## 3 Results

Baseline characteristics of all participants with available data on all DTI measures are shown in Table 1. The mean age at time of MRI was 65.0 years, and 55.8% of the included participants were women. Correlations (Pearson) between the included diffusion measures ranged between 0.46 and 0.85 (Supplemental Fig. S1). While PSMD and MD increased with age, FA declined with increasing age, as expected (Supplemental Fig. S2).

**Table 1.** Characteristic of the total study population.

Characteristic	Total (N = 5230)
Age, y	65.0 (10.9)
Sex, female	2919 (55.8%)
Education	
Primary only	445 (8.5%)
Lower vocational	2000 (38.2%)
Intermediate vocational/higher general	1599 (30.6%)
Higher vocational/university	1186 (22.7%)
Body mass index, kg/m^2^	27.4 (4.1)
Systolic blood pressure, mm Hg	139.8 (21.4)
Diastolic blood pressure, mm Hg	82.9 (11.0)
Blood pressure lowering medication	1893 (36.2%)
Smoking	
Never	1617 (30.9%)
Former	2550 (48.8%)
Current	1063 (20.3%)
Alcohol intake^a^, g/d	6.43 (0.54–8.57)
Total cholesterol, mmol/L	5.52 (1.06)
HDL cholesterol, mmol/L	1.45 (0.42)
Lipid lowering medication	1323 (25.3%)
Diabetes mellitus	672 (12.8%)
*APOE* genotype	
ε3/ε3	2931 (56.0%)
ε2/ε2 or ε2/ε3	864 (16.5%)
ε4-carrier (heterozygous)	1262 (24.1%)
ε4-carrier (homozygous)	173 (3.3%)
Cerebral microbleeds (any)	1064 (20.3%)
Lacunes (any)	421 (8.0%)

a Variable with skewed distribution. Median (interquartile range) is shown instead of mean (SD).

Abbreviations: HDL = high-density lipoprotein; ICV = intracranial volume. Continuous variables are presented as mean (SD) and categorical variables as n (%).

### 3.1 Risk of dementia

During a median follow-up of 9.8 years, 286 participants developed dementia. Those who developed dementia were often female (62.6%) with a mean age of 77.1 years (standard deviation: 7.5). All diffusion imaging markers were significantly associated with risk of dementia (all hazard ratios> = 1.36) (Fig. 2), with the best goodness of fit for models including MD and PSMD (AIC for MD: 4088.8; and for PSMD: 4089.4). These values were better than for global FA, tract-specific MD or tract-specific FA measures (all AIC>4101.1). The model with PSMD achieved a slightly higher C-statistic (0.886 [95%CI: 0.871–0.900]) than models with MD (C-statistic_MD_: 0.882 [0.867–0.897]) and FA (C-statistic_FA_: 0.876 [0.860–0.891]) (Supplemental Table S1).

**Fig. 2.**
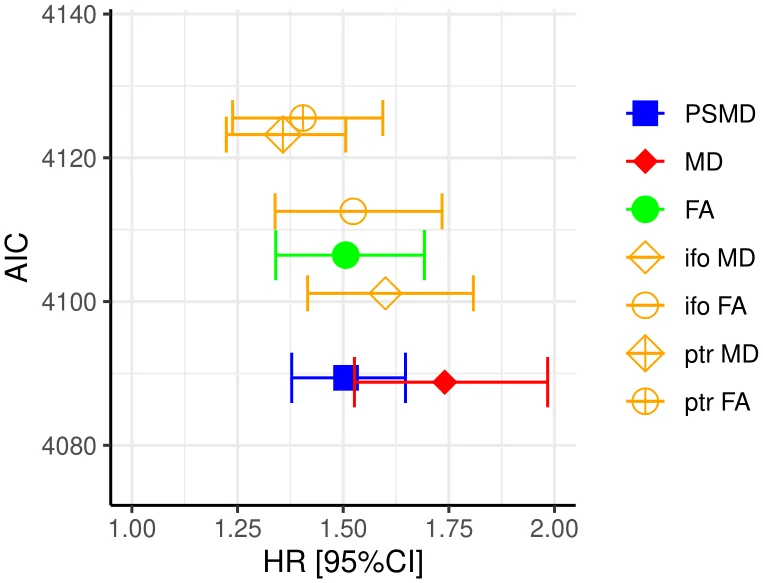
Risk of dementia for diffusion imaging markers. HR=Hazard ratio, CI=confidence interval, AIC=Akaike Information Criterion, PSMD=Peak-width skeletonized mean diffusivity, MD=mean diffusivity, FA=Fractional anisotropy, ifo=inferior fronto-occipital fasciculus, ptr=posterior thalamic radiation; lower AIC reflects better model performance.

When stratifying analyses by follow-up period, models including global MD were superior until 5 years of follow-up, after which global MD and PSMD were comparable in terms of AIC (Supplemental Table S2). HRs were consistently somewhat higher for global MD than for other diffusion measures. In a post-hoc analysis, we assessed the C-statistic of a model including both PSMD and MD which achieved a slightly higher C-statistic (0.889 [95%CI: 0.874–0.903]) than PSMD alone (0.886 [0.871–0.900]). Although the hazard ratio of PSMD decreased from 1.51 [95%CI: 1.38–1.65] to 1.35 [95%CI: 1.21–1.50] when adjusting for MD, it still remained significant (Supplemental Table S3). The C-statistic of WMH (0.876 [0.861–0.891]) was not higher than those of PSMD or global MD, but was similar to the C-statistic of global FA. Combining PSMD and WMH resulted in a C-statistic of 0.885 [95%CI: 0.871–0.900], which is similar to PSMD alone.

### 3.2 Cross-sectional cognition

Of all 5230 participants, 4342 (84.3%) completed on the entire cognitive assessment at time of MRI. Compared to participants who did not complete the entire neuropsychological assessment, those who did were on average younger and had a lower burden of vascular risk factors (Supplemental Table S4).

All diffusion imaging measures were significantly associated with global cognition at time of MRI (Fig. 3A). For global cognition, the regression coefficient for PSMD (β [95%CI]: -0.22 [-0.26– -0.19]) was higher than coefficients for global MD (β [95%CI]: -0.13 [-0.16– -0.10]) and global FA (β [95%CI]: -0.10 [-0.12– -0.07]). Regression coefficients of tract-specific diffusion measures were also inferior to those of PSMD. Moreover, the goodness-of-fit was superior for the model including PSMD (AIC: 9700.9), compared to models with other diffusion imaging measures (AIC>9753.7). The regression coefficient and AIC of WMH for cross-sectional (β [95%CI]: -0.12 [-0.14—0.09]; AIC: 9785.4) were similar to those of MD and inferior to those of PSMD.

**Fig. 3.**
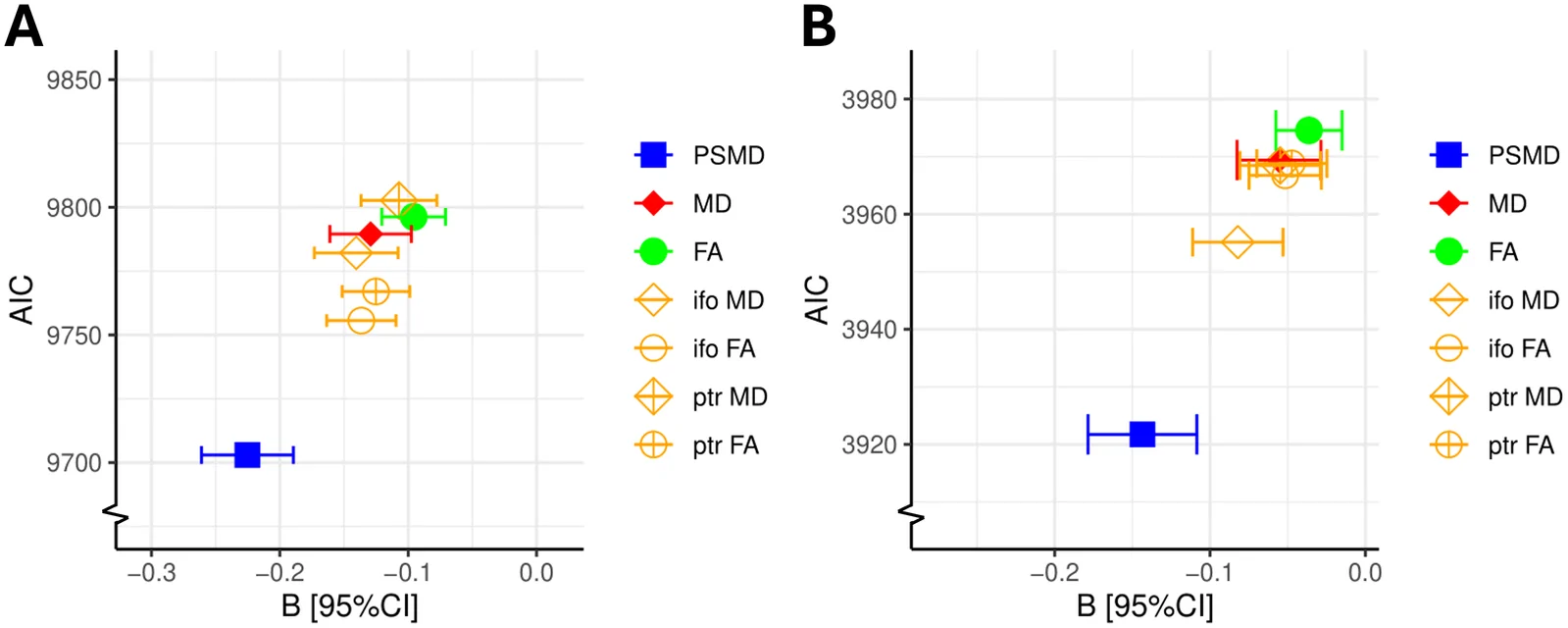
Cognition and diffusion imaging markers. (A) Cross-sectional global cognition (g-factor) and diffusion imaging markers. (B) Longitudinal global cognition (g-factor) and diffusion imaging markers. B=Bèta (regression coefficient), CI=confidence interval, AIC=Akaike Information Criterion, PSMD=Peak-width skeletonized mean diffusivity, MD=mean diffusivity, FA=Fractional anisotropy, ifo=inferior fronto-occipital fasciculus, ptr=posterior thalamic radiation; lower AIC reflects better model performance.

Similarly, for individual cognitive tests, PSMD had the highest regression coefficients and superior model fit, compared to global MD and global FA. Tract-specific diffusion measures showed a superior model fit for the Word Fluency Test and Word Learning Test (Supplemental Fig. S3).

### 3.3 Longitudinal cognition

Of 4342 participants with complete cognitive assessment at baseline, 2800 (64.5%) completed the entire cognitive assessment during reassessment after on average 5.6 years (Supplemental Fig. S4).

All diffusion imaging measures were significantly associated with global cognition (Fig. 3B). The regression coefficient and goodness-of-fit were highest for PSMD (β [95%CI]: -0.14 [-0.18– -0.11]; AIC: 3922.0), compared to MD (β: -0.06 [-0.08– -0.03]; AIC: 3969.6) and FA (β: -0.04 [-0.06– -0.01]; AIC: 3964.8). Tract-specific diffusion measures were also inferior to PSMD regarding global cognition. The regression coefficient and AIC of WMH for longitudinal cognition (β [95%CI]: -0.05 [-0.07– -0.02]; AIC: 3971.1) were similar to those of MD and inferior to those of PSMD.

Consistent with global cognition, PSMD had the highest regression coefficients and superior model fit across all cognitive tests, compared to other global and tract-specific diffusion measures (Supplemental Fig. S5).

## 4 Discussion

In this direct head-to-head comparison of DTI measures in a large population-based imaging study, we demonstrated that both PSMD and global MD are superior for predicting dementia, and PSMD is superior in predicting cognitive decline. PSMD also outperformed tract-specific measures of MD and FA, except for cross-sectional performance in verbal fluency and the 15-word learning test. However, the clinical relevance of this difference remains to be determined.

Our study confirms earlier associations of PSMD with cognitive decline (Deary et al., 2019; Hu et al., 2025; Lam et al., 2019; Luckey et al., 2024) and dementia risk (Hu et al., 2025; Jacob et al., 2023), and adds the insight that these associations for PSMD are at least equal, if not stronger, than for other diffusion imaging measures. This confirms our hypothesis that PSMD may be more sensitive to microstructural damage of cerebral white matter. Based on this rationale, PSMD is expected to be particularly effective in predicting dementia or cognitive decline over longer follow-up periods. This was supported by our results on cross-sectional cognition and cognition at follow-up. In cross-sectional analyses, tract-specific measures outperformed PSMD in the Word Fluency Test and the Word Learning Test. However, when assessing cognitive decline during follow-up, PSMD was stronger than other diffusion imaging markers for all cognitive tests. Furthermore, while models including global MD had the best fit for dementia at follow-up up to 5 years, models including PSMD caught up and showed comparable model fit at longer follow-up durations.

We additionally compared diffusion markers with the conventional structural MRI marker WMH burden. Although WMH was associated with dementia risk and cognitive performance, PSMD consistently showed superior performance, while WMH performed similarly to global MD. Moreover, adding WMH to PSMD did not meaningfully improve prediction of dementia, suggesting that PSMD captures prognostic information beyond conventional structural MRI.

Tract-specific diffusion measures were inferior to PSMD regarding predicting general cognition or dementia, but performed well in cross-sectional analyses of specific cognitive domains. Particularly for the Word Fluency Test and the Word learning Test, the models including the global FA or MD value of the inferior fronto-occipital fasciculus were superior, which can be attributed to the important role this structure has in language processing (Conner et al., 2018). For other cognitive tests, PSMD was superior, which is in line with previous clinic-based and population-based studies, which showed that PSMD is cross-sectionally associated with processing speed, executive function, visuospatial ability, verbal memory, and general cognition (Akbari et al., 2020; Frey et al., 2021; Lam et al., 2019). Moreover, one of these studies showed that PSMD is significantly associated with processing speed and memory at 3-year follow-up (Lam et al., 2019). Although our follow-up for cognitive decline was longer, these associations were also seen in our results regarding individual cognitive tests at follow-up (Supplemental Fig. S5). In the current study, PSMD had the strongest association with all cognitive tests at follow-up, compared to other global and tract-specific diffusion measures.

In addition to being a strong predictor for cognitive decline and dementia, PSMD offers several strengths over other diffusion imaging measures. Both global FA and MD measures may be affected by crossing fibers. These measures aggregate FA or MD values across the entire normal-appearing white matter, making them more susceptible to noise or artefacts. In contrast, PSMD offers greater robustness due to its skeletonized approach, which simplifies the white matter structure into a more focused representation of the principal fiber directions, effectively reducing the impact of crossing fibers. Furthermore, PSMD uses the 5th to 95th percentile of the MD histogram, which helps to minimize the influence of outliers and partial volume with cerebrospinal fluid, providing a more reliable measure of tissue integrity. Consequently, previous research showed that PSMD has excellent inter-rater reliability (between different sites), test-retest repeatability, and inter-scanner reproducibility (Maillard et al., 2022). Unlike voxel-wise approaches such as TBSS, which aim to localize spatially consistent effects, PSMD captures inter-regional heterogeneity of white matter microstructure, making it particularly sensitive to early, focal, and spatially heterogeneous damage that may not survive mass-univariate correction. These characteristics may explain why PSMD consistently outperformed WMH, as diffusion MRI can detect subtle microstructural abnormalities that are not yet visible as macroscopic white matter lesions on conventional structural MRI. The robustness and automated extraction of PSMD (Baykara et al., 2016) along with its predictive value in the current study, may favour PSMD as the preferred measure of structural white matter integrity in prediction, and early diagnosis of cognitive decline and dementia. Although DTI has not been widely adopted in clinical practice yet, our comparative evidence may guide the implementation of the most appropriate diffusion measures for prediction of cognitive decline and dementia.

Strengths of our study include the long-term, prospective ascertainment of dementia and cognition in a large population-representative sample with diffusion brain MRI. However, it is important to acknowledge some methodological limitations. First, excluding participants with incomplete cognitive test data potentially introduced some selection bias. Participants excluded from cross-sectional cognition analysis were a bit older with more vascular risk factors than those with complete data on all cognitive tests (Supplemental Table S1). Second, although >80% of individuals who attended the study center also underwent brain MRI, the selection may have led to a healthier study population, potentially attenuating our estimates but not affecting the comparison between markers. Thirdly, our study was limited to a field strength of 1.5T for the MRI scans. Although higher field strengths provide increased signal-to-noise ratio, there is no clear evidence that moving from 1.5T to higher field strengths affects global FA, global MD or PSMD differentially, particularly when identical preprocessing and analysis pipelines are applied. Fourth, our findings were not validated in other populations or MRI protocols, which future research should address. Last, our study population predominantly consisted of White participants, which may limit the generalizability of the findings to other ethnic groups.

In conclusion, in our head-to-head comparison of structural connectivity measures, PSMD is superior to MD and FA for prediction of cognition and risk of cognitive decline, while both PSMD and global MD are superior with respect to dementia risk. Given its robustness, PSMD may be the preferred imaging marker for predicting cognitive decline in clinical practice.

## Supplementary Material

Supplementary Material

## Data Availability

Requests for the anonymized data underlying this report can be directed to data manager Frank J.A. van Rooij (secretariat.epi@erasmusmc.nl).
