# Supplementary material for: Head-to-head comparison of diffusion imaging markers for cognitive decline and risk of dementia: The population-based Rotterdam Study
Source: Imaging Neurosci (Camb). 2026 Oct 6;4:IMAG.a.1382. doi: 10.1162/IMAG.a.1382 (PMC13644046; doi:10.1162/IMAG.a.1382)
Supplement: Supplementary file 1 [file IMAG.a.1382_supp.pdf]

5.6 Supplementary Materials

Supplemental Table 1 Discriminatory power regarding dementia prediction when using different diffusion measures

	PSMD	MD	FA	Ifo MD	Ifo FA	Ptr MD	Ptr FA
C-statistic [95%CI]	0.886 [0.871-0.900]	0.882 [0.867-0.897]	0.876 [0.860-0.891]	0.881 [0.866-0.896]	0.877 [0.862-0.893]	0.875 [0.860-0.890]	0.876 [0.860-0.891]

PSMD=Peak-width skeletonized mean diffusivity, MD=mean diffusivity, FA=Fractional anisotropy, ifo=inferior fronto-occipital fasciculus, ptr=posterior thalamic radiation; higher C-statistic reflects a stronger discriminatory power.

Supplemental Table 2 Risk of dementia for diffusion imaging markers with increasing follow-up time.

Follow-up	At risk/Cumulative dementia	PSMD		MD		FA		ifo MD		Ifo FA		Ptr MD		Ptr FA	
		HR [95%CI]	AIC	HR [95%CI]	AIC	HR [95%CI]	AIC	HR [95%CI]	AIC	HR [95%CI]	AIC	HR [95%CI]	AIC	HR [95%CI]	AIC
3 years	4968/55	1.38 [1.15-1.67]	833.2	1.96 [1.49-2.57]	**821.4**	1.41 [1.10-1.81]	835.7	1.60 [1.25-2.05]	830.0	1.45 [1.10-1.91]	836.0	1.30 [1.04-1.62]	837.8	1.32 [1.00-1.74]	838.8
4 years	4847/79	1.48 [1.27-1.72]	1183.0	1.90 [1.52-2.39]	**1175.3**	1.47 [1.19-1.81]	1190.8	1.71 [1.40-2.10]	1179.3	1.55 [1.23-1.96]	1189.9	1.39 [1.16-1.67]	1191.8	1.44 [1.14-1.81]	1193.9
5 years	4652/108	1.45 [1.27-1.65]	1592.9	1.78 [1.46-2.17]	**1587.5**	1.35 [1.13-1.62]	1606.6	1.65 [1.38-1.97]	1589.5	1.51 [1.24-1.85]	1601.0	1.41 [1.21-1.65]	1600.5	1.48 [1.21-1.81]	1602.5
6 years	4417/151	1.53 [1.38-1.71]	**2212.6**	1.83 [1.55-2.17]	2213.5	1.47 [1.26-1.71]	2235.4	1.66 [1.43-1.93]	2220.3	1.57 [1.33-1.87]	2232.0	1.36 [1.19-1.56]	2241.1	1.50 [1.26-1.78]	2237.7
7 years	4151/179	1.49 [1.34-1.65]	**2623.5**	1.73 [1.48-2.04]	2624.1	1.37 [1.19-1.58]	2648.0	1.58 [1.37-1.83]	2631.2	1.50 [1.28-1.76]	2641.5	1.31 [1.16-1.49]	2650.4	1.39 [1.19-1.62]	2649.9
8 years	3729/206	1.49 [1.35-1.64]	**3018.5**	1.72 [1.48-2.00]	3019.9	1.43 [1.25-1.63]	3040.1	1.61 [1.41-1.84]	3024.1	1.57 [1.36-1.82]	3031.4	1.34 [1.19-1.50]	3046.2	1.42 [1.23-1.65]	3044.8
9 years	3157/243	1.51 [1.37-1.65]	3555.8	1.78 [1.55-2.05]	**3552.5**	1.46 [1.29-1.66]	3578.1	1.62 [1.42-1.84]	3564.6	1.59 [1.38-1.82]	3570.7	1.36 [1.21-1.51]	3587.0	1.41 [1.23-1.62]	3588.6
10 years	2502/264	1.51 [1.38-1.65]	**3820.4**	1.73 [1.51-1.99]	3822.2	1.48 [1.31-1.67]	3841.6	1.60 [1.42-1.82]	3831.7	1.55 [1.36-1.77]	3840.4	1.34 [1.20-1.49]	3856.1	1.40 [1.23-1.59]	3856.6
11 years	1751/278	1.51 [1.38-1.65]	**3991.3**	1.73 [1.51-1.97]	3992.9	1.50 [1.33-1.68]	4009.8	1.59 [1.40-1.80]	4004.7	1.53 [1.34-1.74]	4014.0	1.34 [1.21-1.49]	4027.4	1.40 [1.23-1.59]	4027.4

12 years	1046/285	1.51 [1.38-1.65]	4076.5	1.75 [1.53-1.99]	**4075.3**	1.51 [1.34-1.69]	4093.4	1.60 [1.41-1.81]	4088.5	1.51 [1.33-1.72]	4101.0	1.36 [1.22-1.51]	4110.5	1.41 [1.24-1.59]	4112.6

HR=Hazard ratio, CI=confidence interval, AIC=Akaike Information Criterion, PSMD=Peak-width skeletonized mean diffusivity, MD=mean diffusivity, FA=Fractional anisotropy, ifo=inferior fronto-occipital fasciculus, ptr=posterior thalamic radiation; lower AIC reflects better model performance. For each follow-up length, the AIC in bold is the optimal (lowest) AIC, compared to those of other diffusion measures.

Supplemental Table 3 Hazard ratios of PSMD for dementia risk, with and without adjusting for MD

	Dementia HR [95%CI]
PSMD*	1.51 [1.38-1.65]
PSMD (additionally adjusted for MD)*	1.35 [1.21-1.50]

Adjusted for age, sex, education, lacunar infarcts, and microbleeds.

HR=hazard ratio, CI=confidence interval, PSMD=peak-width mean diffusivity, MD=mean diffusivity

Supplemental Table 4 Characteristics of the population included in cognition analyses

Characteristic	Participants included in cross-sectional cognition analysis (N=4342)	Participants excluded from cross-sectional cognition analysis due to incomplete data on all cognitive tests (N=853)
Age, y	64.1 (10.7)	69.3 (11.1)
Sex, female	2436 (56.1%)	465 (54.5%)
Education		
Primary only	335 (7.7%)	101 (11.8%)
Lower vocational	1659 (38.2%)	324 (38.0%)
Intermediate vocational/higher general	1330 (30.6%)	266 (31.2%)
Higher vocational/university	1018 (23.4%)	162 (19.0%)
Body mass index, kg/m^2	27.4 (4.1)	27.7 (4.2)
Systolic blood pressure, mm Hg	139.0 (21.2)	143.7 (22.5)
Diastolic blood pressure, mm Hg	82.8 (10.9)	83.3 (11.8)
Blood pressure lowering medication	1473 (33.9%)	402 (47.1%)
Smoking		
Never	1337 (30.8%)	270 (31.7%)
Former	2126 (49.0%)	408 (47.8%)
Current	879 (20.2%)	175 (20.5%)
Alcohol intake[a], g/d	6.43 (0.54-10.00)	6.43 (0.54-8.57)
Total cholesterol, mmol/L	5.54 (1.06)	5.44 (1.07)
HDL cholesterol, mmol/L	1.45 (0.42)	1.45 (0.42)
Lipid lowering medication	1059 (24.4%)	257 (30.1%)
Diabetes mellitus	519 (12.0%)	148 (17.4%)
APOE genotype		
ε3/ε3	2426 (55.9%)	492 (57.7%)
ε2/ε2 or ε2/ε3	707 (16.3%)	143 (16.8%)
ε4-carrier (heterozygous)	1067 (24.6%)	188 (22.0%)
ε4-carrier (homozygous)	142 (3.3%)	30 (3.5%)
Cerebral microbleeds (any)	844 (19.4%)	213 (25.0%)
Lacunes (any)	326 (7.5%)	90 (10.6%)

Abbreviations: HDL = high-density lipoprotein; ICV = intracranial volume. Continuous variables are presented as mean (SD) and categorical variables as n (%). [a] Variable with skewed distribution. Median (interquartile range) is shown instead of mean (SD).

Supplemental Figure 1: Correlation plot of diffusion imaging markers

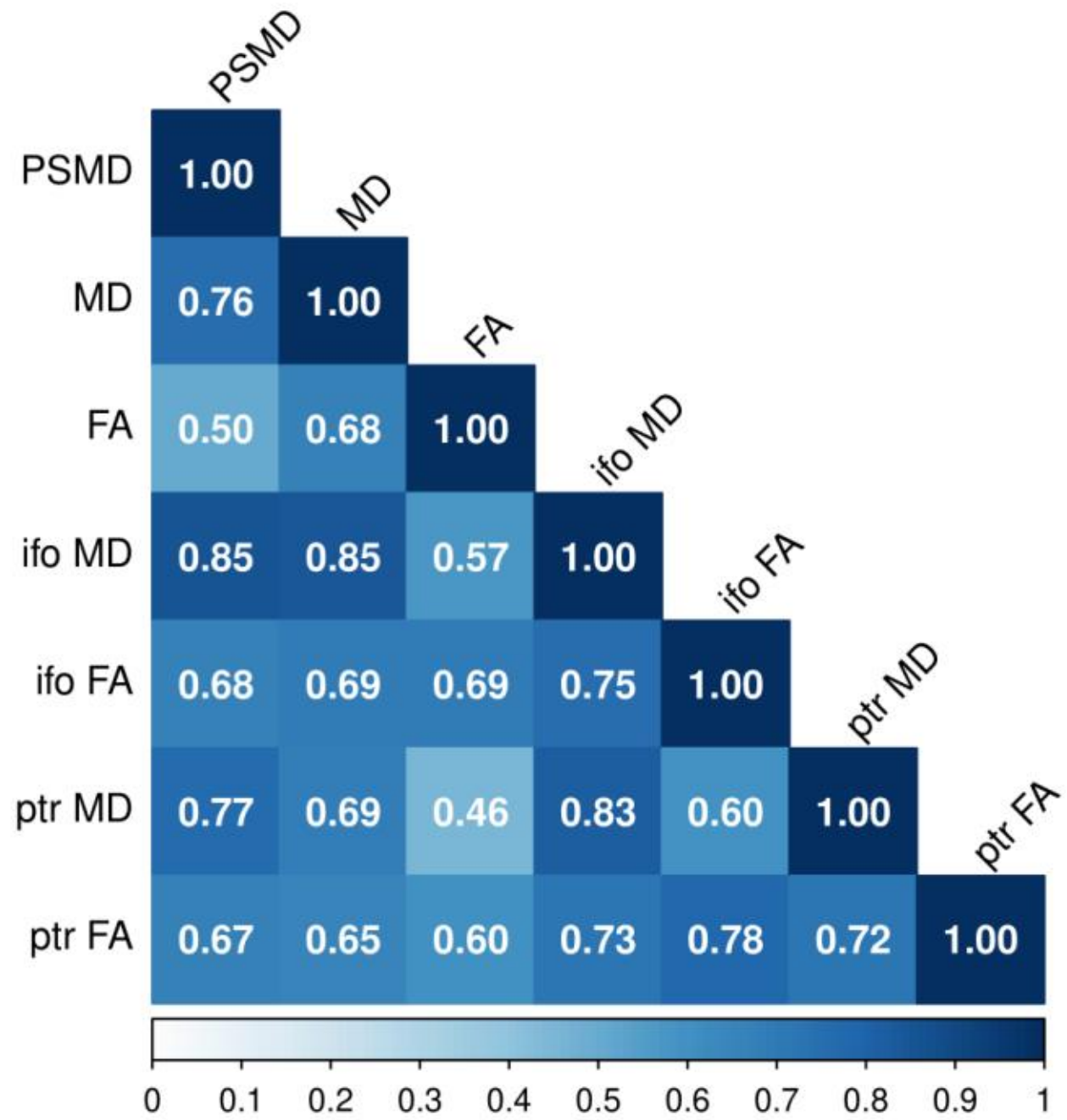

PSMD=Peak-width skeletonized mean diffusivity, MD=mean diffusivity, FA=Fractional anisotropy, ifo=inferior fronto-occipital fasciculus, ptr=posterior thalamic radiation.

Supplemental Figure 2: Scatter plots showing the relationship between diffusion imaging markers (PSMD/MD/FA) and age.

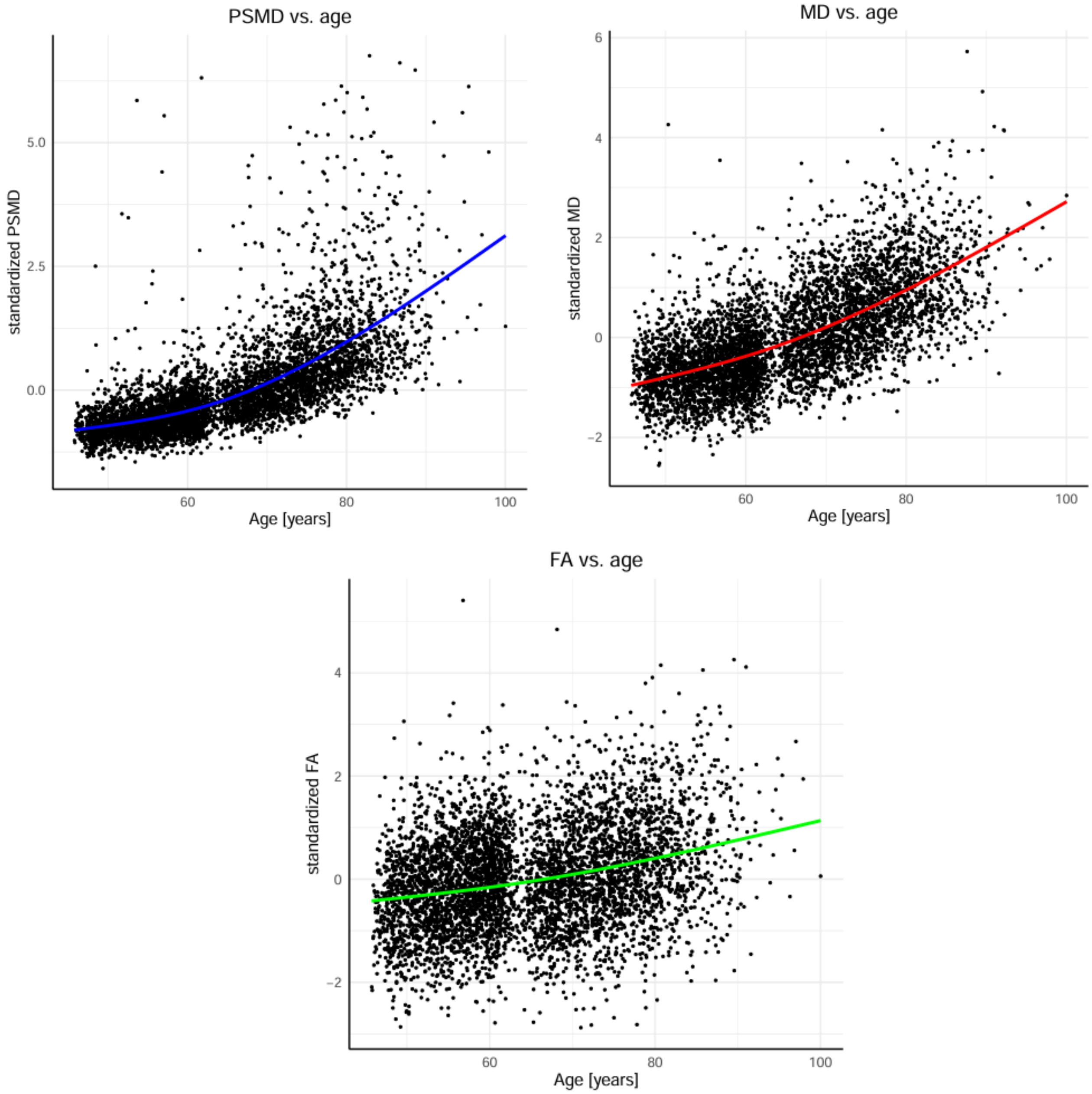

PSMD=Peak-width skeletonized mean diffusivity, MD=mean diffusivity, FA=Fractional anisotropy

Supplemental Figure 3: Cross-sectional cognition and diffusion imaging markers for each cognitive test separately

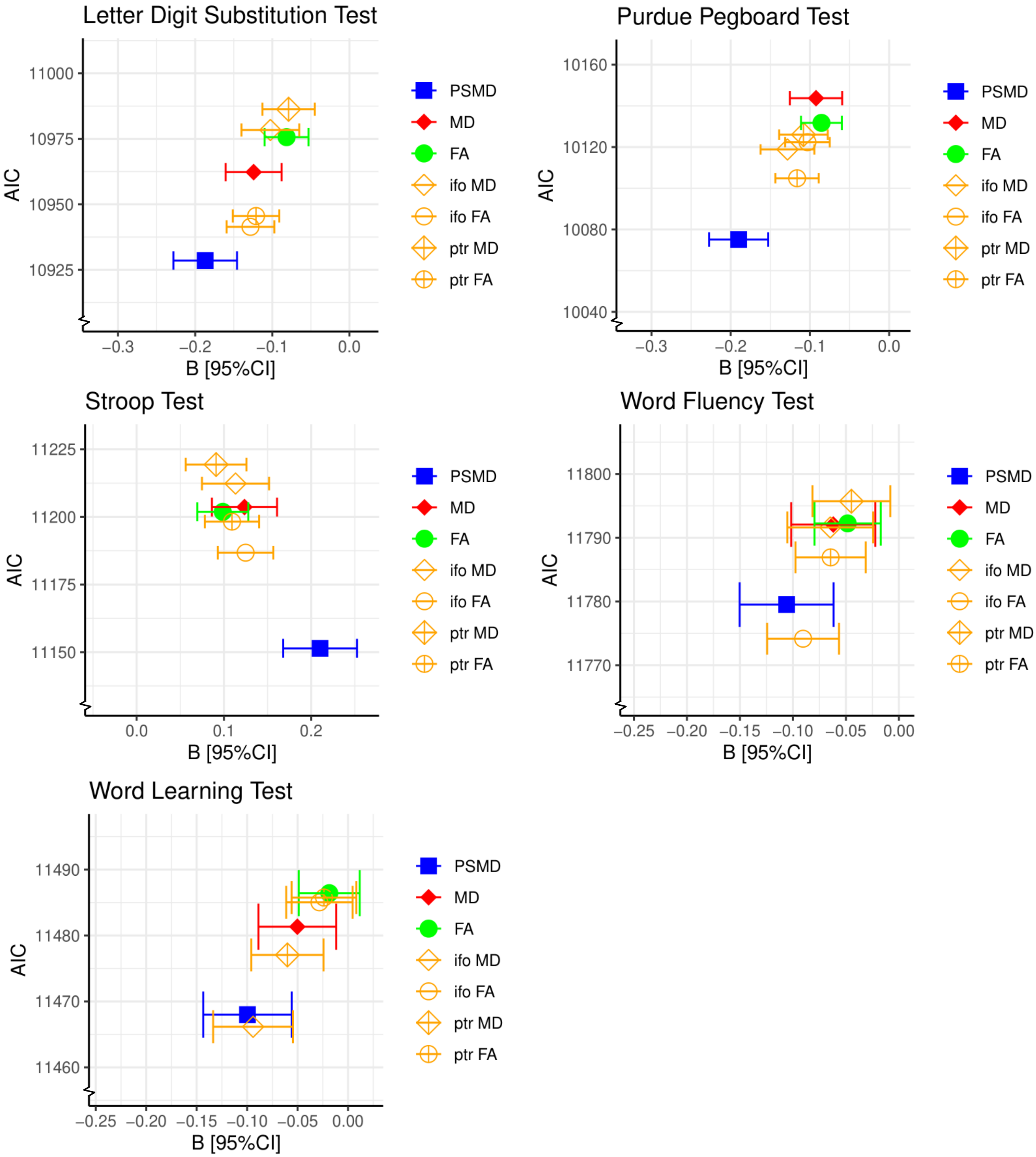

B=Bèta (regression coefficient), CI=confidence interval, AIC=Akaike Information Criterion, PSMD=Peak-width skeletonized mean diffusivity, MD=mean diffusivity, FA=Fractional anisotropy, ifo=inferior fronto-occipital fasciculus, ptr=posterior thalamic radiation; lower AIC reflects better model performance.

Supplemental Figure 4: Histogram of follow-up time between MRI and cognitive performance measured at next follow-up visit.

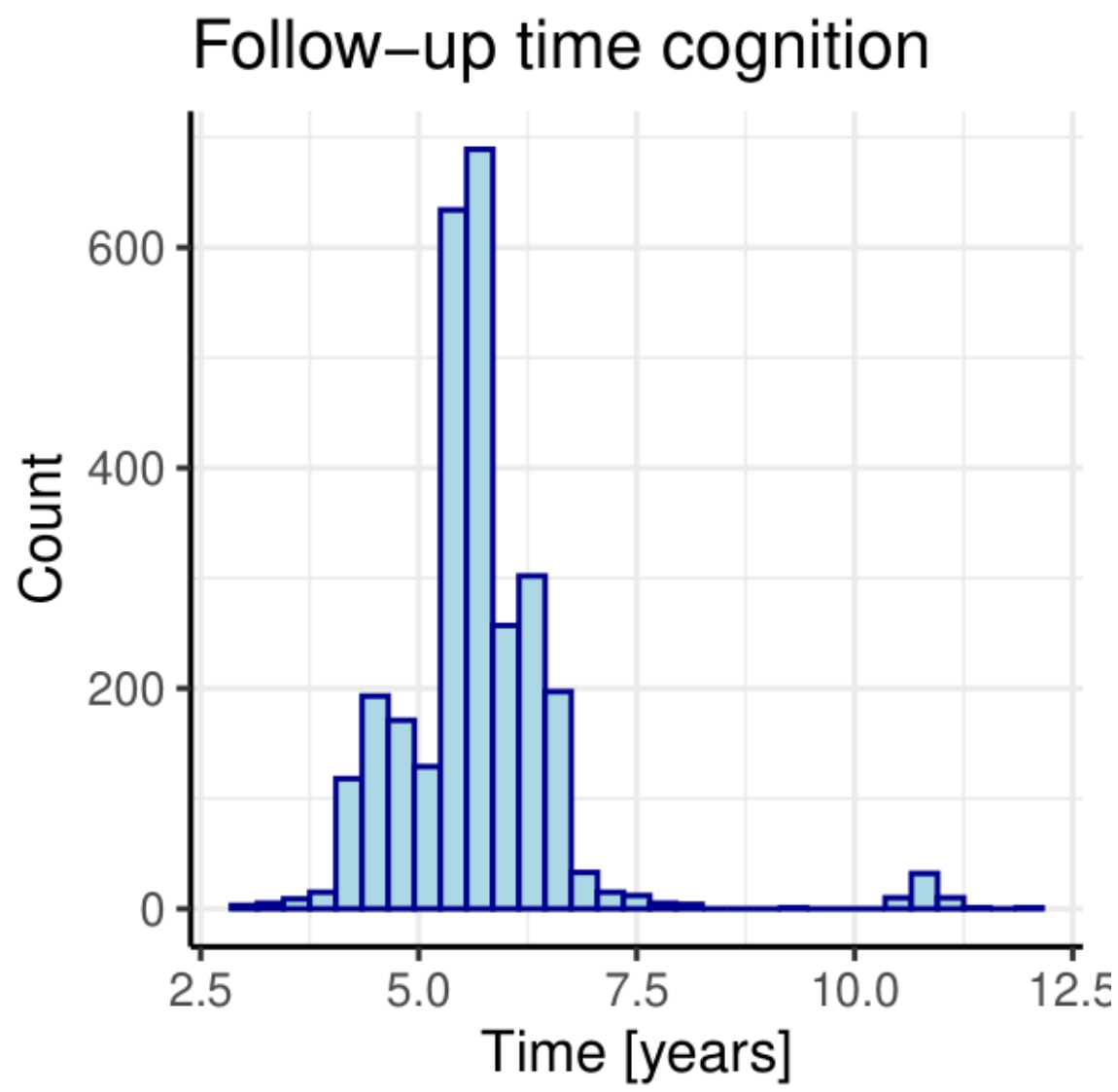

Supplemental Figure 5: Longitudinal cognition and diffusion imaging markers for each cognitive test separately

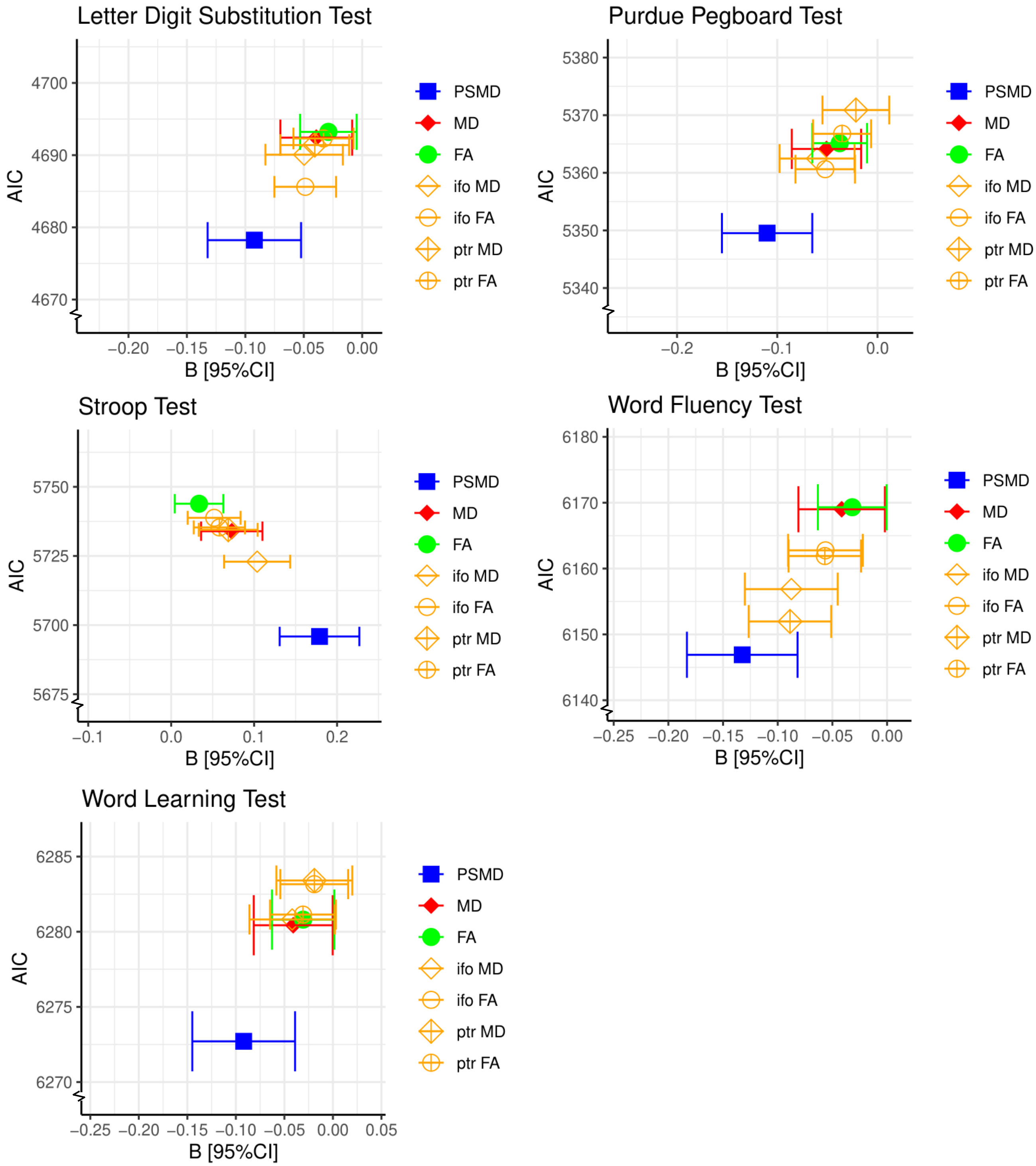

B=Bèta (regression coefficient), CI=confidence interval, AIC=Akaike Information Criterion, PSMD=Peak-width skeletonized mean diffusivity, MD=mean diffusivity, FA=Fractional anisotropy, ifo=inferior fronto-occipital fasciculus, ptr=posterior thalamic radiation; lower AIC reflects better model performance.
